# Cost-Effectiveness Analysis of Risk-Factor Guided and Birth-Cohort Screening for Chronic Hepatitis C Infection in the United States

**DOI:** 10.1371/journal.pone.0058975

**Published:** 2013-03-22

**Authors:** Shan Liu, Lauren E. Cipriano, Mark Holodniy, Jeremy D. Goldhaber-Fiebert

**Affiliations:** 1 Department of Management Science and Engineering, Stanford University, Stanford, California, United States of America; 2 Veterans Affairs Palo Alto Health Care System, Palo Alto, California, United States of America; 3 Stanford Health Policy, Centers for Health Policy and Primary Care and Outcomes Research, Stanford University, Stanford, California, United States of America; University of Modena & Reggio Emilia, Italy

## Abstract

**Background:**

No consensus exists on screening to detect the estimated 2 million Americans unaware of their chronic hepatitis C infections. Advisory groups differ, recommending birth-cohort screening for baby boomers, screening only high-risk individuals, or no screening. We assessed one-time risk assessment and screening to identify previously undiagnosed 40–74 year-olds given newly available hepatitis C treatments.

**Methods and Findings:**

A Markov model evaluated alternative risk-factor guided and birth-cohort screening and treatment strategies. Risk factors included drug use history, blood transfusion before 1992, and multiple sexual partners. Analyses of the National Health and Nutrition Examination Survey provided sex-, race-, age-, and risk-factor-specific hepatitis C prevalence and mortality rates. Nine strategies combined screening (no screening, risk-factor guided screening, or birth-cohort screening) and treatment (standard therapy–peginterferon alfa and ribavirin, Interleukin-28B-guided (IL28B) triple-therapy–standard therapy plus a protease inhibitor, or universal triple therapy). Response-guided treatment depended on HCV genotype. Outcomes include discounted lifetime costs (2010 dollars) and quality adjusted life-years (QALYs).

Compared to no screening, risk-factor guided and birth-cohort screening for 50 year-olds gained 0.7 to 3.5 quality adjusted life-days and cost $168 to $568 per person. Birth-cohort screening provided more benefit per dollar than risk-factor guided screening and cost $65,749 per QALY if followed by universal triple therapy compared to screening followed by IL28B-guided triple therapy. If only 10% of screen-detected, eligible patients initiate treatment at each opportunity, birth-cohort screening with universal triple therapy costs $241,100 per QALY. Assuming treatment with triple therapy, screening all individuals aged 40–64 years costs less than $100,000 per QALY.

**Conclusions:**

The cost-effectiveness of one-time birth-cohort hepatitis C screening for 40–64 year olds is comparable to other screening programs, provided that the healthcare system has sufficient capacity to deliver prompt treatment and appropriate follow-on care to many newly screen-detected individuals.

## Introduction

An estimated 2 million Americans are unaware that they are infected with hepatitis C (HCV) [1]. Without diagnosis and treatment, they are at risk for liver fibrosis, cirrhosis, and hepatocellular-carcinoma (HCC). HCV-caused end-stage liver disease is the leading cause of liver transplantation {Ly, #366}. The prevalence of HCV antibodies is approximately 5% in individuals born between 1950 and 1960, over twice the general adult prevalence [3]. Lifestyle factors (e.g., history of injection drug use, blood transfusion before 1992, and risky sexual behaviors) are predictive of HCV infection, though not everyone is willing to divulge their true risk status to their clinicians. Screening – whether risk-based or birth-cohort-based – could potentially prevent substantial HCV-related losses of health and life provided those identified via screening receive appropriate treatment. Given the large number of screen-eligible individuals, it is important to determine which screening strategy is most cost-effective.

The CDC recently recommended one-time screening for all individuals born between 1945 and 1965 [4]. Previously, the National Institutes of Health Consensus Panel [5] and the American Liver Foundation [6] recommend screening only high-risk individuals. In 2004, the U.S. Preventive Services Task Force (USPSTF) recommended against birth-cohort HCV screening and found that the evidence supporting screening high-risk individuals was insufficient [7]. In 2012, the USPSTF produced a draft recommendation on screening for HCV infection in high-risk adults including those with any history of intravenous drug use or blood transfusions prior to 1992, which it is currently updating [8].

HCV screening guidelines require reconsideration in light of new, more effective, treatments [9], [10], potentially combined with patient genotyping (Interleukin (IL)–28B) to personalize treatment selection. The effectiveness and cost-effectiveness of HCV screening policies will depend on screening-related factors which determine how many additional people could be identified (e.g., prevalence of undiagnosed HCV infections; the predictive power of HCV risk factors) and treatment-related factors which determine how much benefit can be delivered and at what cost for each person identified (e.g., access to and choice of treatment).

Prior studies have evaluated the cost-effectiveness of birth-cohort screening compared to risk-based screening, though none have simultaneously included a number of important clinical and epidemiological considerations. A 2001 study did not support universal HCV screening among asymptomatic, average-risk American adults [11]. More recent studies found that birth-cohort screening costs between $5,400 and $37,700 per QALY gained [12], [13], [14]. No study has simultaneously compared risk-factor guided screening to birth-cohort screening, explicitly modeling risk assessment to identify high-risk individuals; included mortality differences between risk groups, whose exclusion may bias towards the cost-effectiveness of screening; and considered how the cost-effectiveness of screening depends on the quality of follow-up care, treatment uptake and adherence for the many screen-detected individuals.

We assessed the cost-effectiveness of one-time screening of 40–74 year-olds at a routine medical visit, addressing two questions: 1) Can costs and benefits of screening be improved by using risk assessment to identify asymptomatic individuals who are more likely HCV infected? 2) How is the cost-effectiveness of HCV screening affected by subsequent disease management, HCV treatment uptake, and treatment type?

## Methods

### Cohorts

The decision-analytic model applies screening and treatment strategies to asymptomatic 40–74 year-old (base case age 50) U.S. adults who are unaware of their HCV infection status, with attention focused on how treatment uptake and ongoing HCV care affect outcomes. Cohorts are stratified by age, sex, race, risk history, HCV infection status, HCV genotype, treatment eligibility, IL-28B genotype, and initial liver fibrosis stage. Model inputs are presented in Table 1.

**Table 1:** Model Parameter Values and Ranges.

Variable	Base Case (Range)	Reference
**Model assumptions**		
Discount rate (annual)	0.03 (0.00–0.05)	[41]
Time horizon	Lifetime	
Perspective	Societal	
**Cohort characteristics**		
Cohort age, ***years***	50 (40–74)	
Stage of fibrosis distribution in HCV+ population		[17]
No fibrosis (F0)	0.13	
Portal fibrosis (F1)	0.51	
Periportal fibrosis (F2)	0.13	
Bridging fibrosis (F3)	0.10	
Compensated fibrosis (F4)	0.13	
Proportion with HCV genotype 1	0.8 (0.7–0.9)	[5]
Proportion with IL-28B genotype, CC-type polymorphism (vs. non–CC type)		
White	0.37 (0.28–0.46)	[46]
Black	0.14 (0.11–0.18)	
**Risk Status (by sex, race)** *		NHANES 2001–2008
Percent of high risk individuals (%)		
White male	26 (24–28)	
White female	15 (13–18)	
Black male	31 (28–35)	
Black female	19 (17–22)	
Prevalence of HCV+ among high-risk individuals (%)		
White male	13 (10–16)	
White female	11 (8–15)	
Black male	17 (13–21)	
Black female	15 (11–19)	
Prevalence of HCV+ among low-risk individuals (%)		
White male	2 (1–3)	
White female	2 (1–2)	
Black male	3 (2–4)	
Black female	2 (1–3)	
Awareness of HCV status (%)		NHANES 2001–2008
Percent aware among HCV+ high-risk individuals	50 (5–60)	
Percent aware among HCV+ low-risk individuals	50 (0–60)	
Percent aware among HCV- high-risk individuals	50 (0–60)	Assumed
Percent aware among HCV- low-risk individuals	5 (0–10)	Assumed
Annual probability of chance identification of HCV+	0.037 (0.010–0.050)	[11]
**Screening characteristics**		
Risk Identification		
Probability of identified as “high-risk” among true high-risk individuals (sensitivity)		[24]
Male	0.58 (0.00–1.00)	
Female	0.69 (0.00–1.00)	
Probability of identified as “low-risk” among true low-risk individuals (specificity)	1	Assumed
HCV screening test (ELISA)		
Probability of test+among HCV+ (sensitivity)	0.970 (0.950–0.999)	[47]
Probability of test - among HCV- (specificity)	0.9996 (0.9900–1.000)	[48]
HCV natural history		
Proportion of patients with no fibrosis (F0) who do not progress	0.24 (0.20–0.33)	[16]
Annual probability of spontaneous remission from no fibrosis (F0) health state	0.012 (0.007–0.017)	[16], [30]
Fibrosis progression (annual probability)		[16], [30]
Males		
Age 40–49 y	0.05 (0.03–0.09)	
Age 50–59 y	0.12 (0.07–0.14)	
Age 60–69 y	0.20 (0.12–0.30)	
Age ≥70 y	0.26 (0.14–0.38)	
Females		
Age 40–49 y	0.03 (0.01–0.06)	
Age 50–59 y	0.06 (0.03–0.11)	
Age 60–69 y	0.11 (0.04–0.21)	
Age 70–79 y	0.14 (0.08–0.24)	
Age ≥80 y	0.20 (0.08–0.30)	
Cirrhosis to decompensated cirrhosis	0.04 (0.03–0.05)	
Cirrhosis (both F4 and decompensated cirrhosis) to HCC	0.02 (0.017–0.03)	
Liver transplant (annual probability)		[49]
Decompensated cirrhosis to liver transplant	0.05 (0.00–0.40)	
HCC to liver transplant	0.15 (0.05–0.40)	
Chronic HCV conversion factor		NHANES 2001–2008
Male	0.72 (0.58–0.89)	
Female	0.65 (0.60–0.70)	
Hazard ratio for sex-, race-, risk-, HCV-, and age-specific mortality from non-liver causes in patients with chronic HCV infection (< age 70)	Appendix S1 Table S1	NHANES III
Reduction factor on background mortality after successful treatment^&^	0.7 (0.3–1.0)	[22]
Liver-related mortality (annual probability)		
Liver transplant	0.140 (0.134–0.150)	[50]
After liver transplant	0.050 (0.049–0.051)	[50]
Decompensated cirrhosis	0.26 (0.12–0.33)	[16]
HCC		[51]
First year	0.72 (0.58–0.80)	
Subsequent year	0.25 (0.16–0.30)	
Treatment-related mortality	0.0050 (0.0005–0.0110)	[52]
Liver biopsy-related mortality	0.0003 (0.0000–0.0033)	[53]
Probability of FibroTest showing F2+ for patients in F0–F1 Fibrosis	0.13 (0.06–0.15)	[27]
**Treatment characteristics**		
Percent of treatment eligible among diagnosed HCV+	0.86 (0.75–0.95)	[25]
Percentage of people accepting treatment when offered (%)		[25], [26]
Genotype 1, F0–F1 fibrosis	30 (10–90)	
Genotype 1, F2–F4 fibrosis	39 (10–90)	
Genotype 2&3, F0–F1 fibrosis	30 (10–90)	
Genotype 2&3, F2–F4 fibrosis	39 (10–90)	
Effectiveness of treatment in genotype 1 patients	Details in Appendix S1 Table S2	[15]
Standard therapy (PEG-INF+Rb)		[46], [54], [55]
Mild fibrosis (F0/F1/F2), white		
Overall probability of SVR	0.46 (0.42–0.49)	
Mild fibrosis (F0/F1/F2), black		
Overall probability of SVR	0.19 (0.13–0.24)	
Triple therapy (PEG-INF+Rb+PI) **		[9], [56], [57], [58], [59], [60]
Adherence to triple therapy	0.70 (0.50–0.70)	
Mild fibrosis (F0/F1/F2), white		
Overall probability of SVR	0.68 (0.60–0.72)	
Mild fibrosis (F0/F1/F2), black		
Overall probability of SVR	0.42 (0.24–0.47)	
Effectiveness of treatment in genotype 2&3 patients	0.80 (0.60–0.90)	[1], [55]
Reduction in SVR for advanced fibrosis stage (F3 and F4)	0.80 (0.70–1.00)	
Quality of life***		
Age-specific QALY weights		[28], [29]
HCV-specific weights		[30], [31], [32], [33], [34]
HCV mild fibrosis (F0, F1)	0.980 (0.700–1.000)	
SVR after mild fibrosis	1.000 (0.740–1.000)	
HCV moderate fibrosis (F2, F3)	0.850 (0.660–1.000)	
SVR after moderate fibrosis	0.933 (0.710–1.000)	
Compensated cirrhosis (F4)	0.790 (0.460–1.000)	
SVR after cirrhosis	0.933 (0.600–1.000)	
Decompensated cirrhosis	0.720 (0.257–0.913)	
HCC	0.720 (0.150–0.950)	
Liver transplant (during or after)	0.825 (0.636–1.000)	
Standard therapy annualized decrement†	−0.110 (−0.200–0.000)	
Triple therapy annualized decrement†	−0.165 (−0.400–0.000)	
Liver transplant annualized decrement†	−0.200 (−0.364–0.000)	
Liver biopsy decrement^ ˆ^	−0.055 (−0.200–0.000)	
HCV awareness annualized decrement	−0.020 (−0.050–0.000)	[11]
**Cost (2010 U.S. dollars), *$***		
Age-specific baseline health care costs		[36]
Screening		CMS
HCV anti-body screening (ELISA)	20 (10–31)	CPT 86803
Risk identification (HCV+)	36 (18–54)	CPT 99401
Diagnosis (2 confirmatory ELISA, RIBA, and RNA test)	210 (105–315)	2 × (CPT 86803)+CPT 86804+ CPT 87522
Reporting to the patient the results of a negative test	8 (0–11)	[61]
HCV genotyping	369 (184–553)	CPT 87902
IL-28B genotyping	371 (186–557)	[15]
Liver biopsy	1,340 (990–1,650)	CPT 47000
FibroTest	240 (102–300)	[27]
Treatment (drug and medical care)		
PEG-INF+Rb (F0 to F3, 24 wk)	16,346 (6,001–24,730)	[26], [62]
PEG-INF+Rb (F0 to F3, 24 wk)	17,907 (7,562–26,291)	[26], [62]
PEG-INF+Rb (F0 to F3, 48 wk)	32,692 (12,002–49,460)	[26], [62]
PEG-INF+Rb (F4, 48 wk)	35,814 (15,123–52,582)	[26], [62]
PIs (per week)†	1,100 (781–1,430)	[63], [64]
AEs, standard therapy	1,920 (1344–2,496)	[65]
AEs, standard therapy, PI	2,586 (1810–3,361)	[65]
Annual care^||^		[26], [30], [37], [66], [67]
Aware of HCV status		
HCV mild fibrosis (F0, F1)	1,404 (152–4,194)	
HCV portal fibrosis (F2)	1,404 (152–4,194)	
HCV bridging fibrosis (F3)	1,404 (152–4,194)	
Compensated cirrhosis (F4)	4,194 (152–4,194)	
Unaware of HCV status		
HCV mild fibrosis (F0, F1)	811 (0–1,404)	
HCV portal fibrosis (F2)	811 (0–1,404)	
HCV bridging fibrosis (F3)	811 (0–1,404)	
Compensated cirrhosis (F4)	1,622 (0–4,194)	
Decompensated cirrhosis	11,109 (5,560–16,669)	
HCC	44,224 (22,117–66,341)	
Liver transplant, first year	145,640 (72,825–218,455)	
Liver transplant, subsequent	25,430 (12,715–38,156)	
Recovered states from F0 to F3	406 (0–702)	Assumed¶
Recovered states from F4	811 (0–2,097)	Assumed¶

HCC = hepatocellular carcinoma; HCV = hepatitis C virus; IL-28B = interleukin-28B; NHANES III = Third National Health and Nutrition Examination Survey; PEG-IFN = pegylated interferon; PI = protease inhibitor; Rb = ribavirin; SVR = sustained virologic response; AE = adverse event; QALY = quality-adjusted life-year; CMS =  Center for Medicare & Medicaid Services. For further details on parameter generation and the uncertainty distribution of parameters see Appendix S1 I; Appendix S1 I Table S2; Appendix S1 Table S3.

* A high-risk individual is someone having a history of injection drug use, transfusion prior to 1992, or greater than 20 lifetime sex partners. The reported prevalence is estimated for the 1952–1961 birth cohort and include individuals both aware and unaware of their HCV infection status. We adjusted the prevalence to only include individuals unaware of their infection status in the cost-effectiveness analyses.

& The mortality rates for people who recovered from HCV are adjusted by a linear combination of their mortality rates with HCV and mortality rates without HCV using a factor of 0.7.

** The reported triple therapy effectiveness in the base-case is similar to boceprevir.

*** The total quality-of-life weight for a given age and HCV disease state is computed as the product of the mean age-specific quality weight obtained from published data [28], [29] and the utility associated with the HCV disease state minus any utility decrements for events that occurred during the cycle such as receiving treatment or a liver transplant.

† Unlike other utilities in this table, these utility decrements are for short-term states (that is, receiving HCV treatment or a liver transplant). The QALY decrement for receiving HCV treatment involves multiplying the annual utility decrement by the time on treatment, which can vary given the response-guided therapy rules of each strategy. ˆOne time disutility applied in a 12 weeks period.

‡ The PI cost is added to the standard therapy cost while receiving triple therapy.

|| The total costs for a given age and HCV disease state is computed as the sum of the mean age-specific health care costs [36] and the HCV-specific health state plus any costs of testing, treatment, or liver transplant that occurred in the cycle.

¶ We assumed costs in the recovered states are 50% of the hepatitis C–related care costs in the year before diagnosis of the corresponding unaware states [37].

### Screening and Treatment

**Figure 1:**
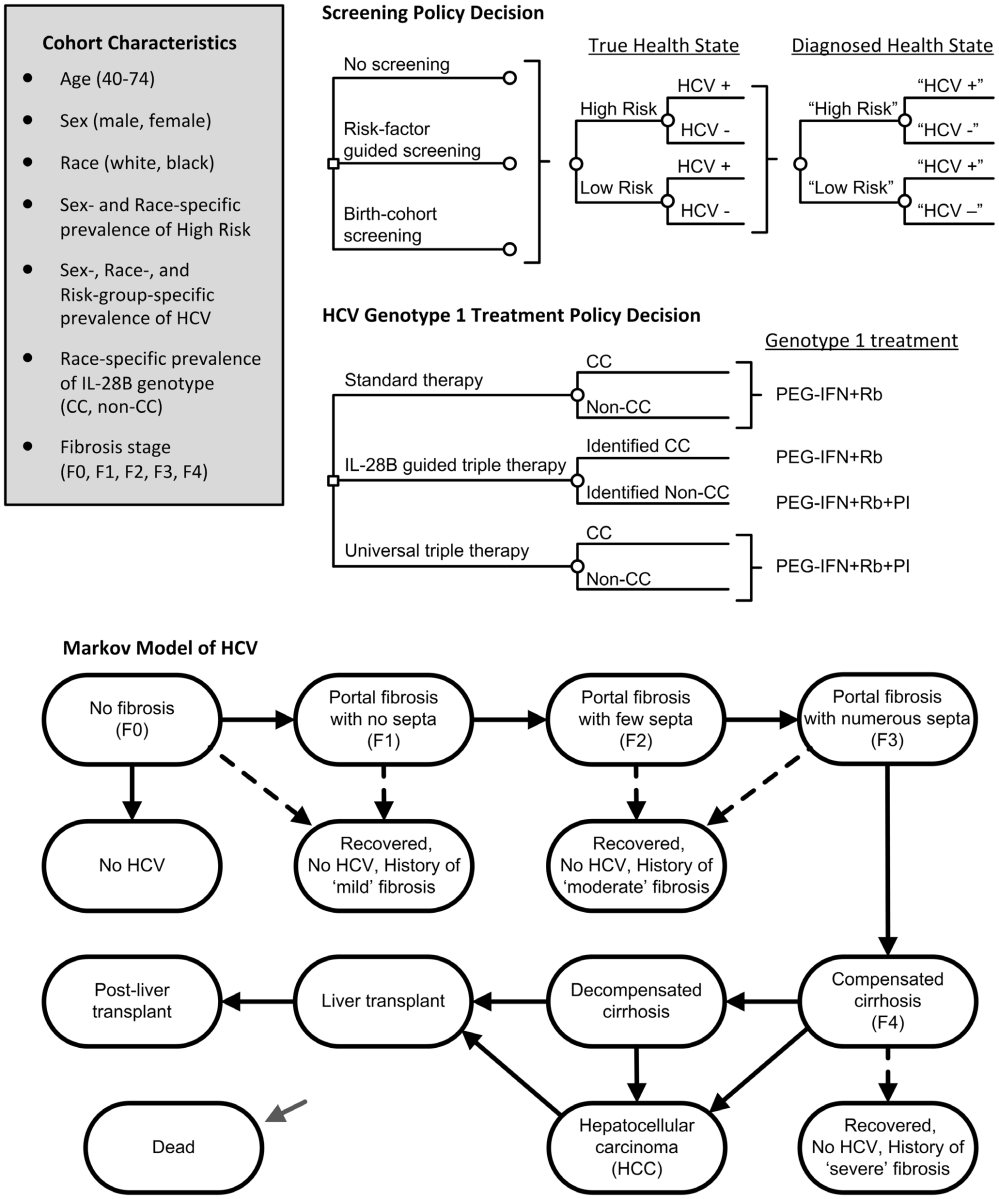
Model schematics. Small squares represent decisions. For the screening policy decision we considered the alternatives of implementing a policy of no screening, risk-factor guided screening, and birth-cohort screening. For the HCV genotype 1 treatment policy decision we considered the alternatives of standard therapy, in which patients receive pegylated interferon with ribavirin; IL-28B-guided triple therapy, in which after IL-28B genotyping patients with non-CC types receive triple therapy and patients with CC types receive standard therapy; and universal triple therapy, in which patients receive pegylated interferon with ribavirin and a protease inhibitor. In all strategies patients diagnosed with genotypes 2 and 3 receive 24 weeks of standard therapy. We considered all possible combinations of the screening policy decision and the genotype 1 treatment policy decision for a total of 9 policy alternatives. Small circles indicate chance events. Upon entering the model the cohort is stratified by true health state of risk-factor status (high risk or low risk), HCV-status (positive or negative), among HCV-positive individuals by HCV genotype (genotype 1 or other), and among HCV-positive genotype 1 individuals by IL-28B genotype (CC or non-CC type). Depending on the screening strategy, individuals may be imperfectly identified as “high-risk” or “low-risk”, may be screened for HCV, and may be imperfectly identified as “HCV+” and “HCV–”. Once individuals are classified with a diagnosis they enter one of two Markov models based on their true health state. The Markov model of HCV is shown. The Markov model of individuals who do not have HCV has only two health states, No HCV and Dead. We assume no HCV incidence in the model. HCC = hepatocellular carcinoma; HCV = hepatitis C virus; IL-28B = interleukin-28B; PEG-IFN = pegylated interferon; PI = protease inhibitor; Rb = ribavirin.

One-time screening is performed at a routine medical visit at the outset of the analysis. We assessed screening strategies in combination with treatment strategies (Figure 1). Screening strategies included: 1) No screening: no systematic screening but HCV infected individuals may receive treatment after chance identification; 2) Risk-factor guided screening: HCV screening is only offered to individuals classified as “high risk” through an imperfect assessment of their risk history; and 3) Birth-cohort screening: all individuals are offered HCV screening. A diagnosis of “HCV-positive” occurs after a positive result on the initial enzyme immunoassay (ELISA) test, confirmed by two ELISAs, a recombinant immunoblot assay (RIBA), and a HCV RNA test to verify chronic infection. Treatment-eligible, chronically-infected individuals have their HCV infection genotyped. Three response-guided treatment strategies for HCV genotype 1 infected patients [15] were: 1) Standard therapy: patients receive pegylated interferon with ribavirin; 2) Universal triple therapy: patients receive pegylated interferon with ribavirin and a protease inhibitor; or 3) IL-28B-guided triple therapy: using IL-28B genotyping, non-CC type patients receive triple therapy and CC type patients receive standard therapy. Standard and triple therapy treatments employ specific response-guided protocols (Table S2 in Appendix S1) [15]. In all cases, patients diagnosed with genotype 2 and 3 receive 24 weeks of standard therapy.

### HCV Natural History

The HCV natural history follows a previously-published empirically-calibrated model [15], [16]. Chronically infected individuals start with an initial distribution of liver fibrosis stages (Metavir scores of F0, F1, F2, F3, or F4) and progress toward advanced liver disease (Figure 1) [17]. Disease progression rates depend on age and sex with possible transitions occurring every 12 weeks. We note that disease progression rates from a meta-analysis by Thein et al. [18] are within range to the values in our model. Successful treatment arrests further progression.

### Risk Factors and HCV Prevalence

“High-risk” was defined as having a history of injection drug use, transfusion prior to 1992, or greater than 20 lifetime sex partners [3]. We estimated the prevalence of risk factors and of HCV among high- and low-risk individuals stratified by age, sex, and race using the National Health and Nutrition Examination Survey (NHANES) (2001–2008) (Section I in Appendix S1).

### Mortality

Mortality rates by age, sex, race, risk status, and HCV infection were calculated using hazard ratios estimated from the NHANES III linked mortality data and U.S. life-tables [19] (Table S1 in Appendix S1). Successful treatment prevents long-term consequences of HCV and reduces non-liver-related mortality, which still remains higher than for individuals with no history of HCV [20], [21], [22], [23]. As age-specific mortality rates of individuals who have recovered from HCV are unknown, we reduced their pre-treatment rates by 0.80 and explored this assumption in sensitivity analyses.

### Risk Assessment

Risk-based screening depends on the ability of healthcare workers to accurately identify high-risk patients. We assumed that assessment of risk-factor status had 100% specificity but a sensitivity of 60% (men) and 70% (woman) [24], varying these widely in sensitivity analyses.

### Treatment Eligibility

Medical contraindications (e.g., medical and psychiatric co-morbidities, active substance abuse, and alcoholism) leave 14% of HCV patients ineligible for treatment [25].

### Treatment Uptake and Ongoing Monitoring

We assumed 30% and 39% of eligible individuals with biopsy-established fibrosis stage F0–F1 and F2–F4, respectively, would initiate treatment immediately [25], [26]. For those who do not, progression surveillance occurs every 3 years using non-invasive fibrosis assessment [27]. We assume that progression to F2 leads 39% of patients with F0–F1 fibrosis at diagnosis to initiate treatment and that 39% of treatment-eligible patients with F2–F4 fibrosis will initiate treatment every three years.

### Health-Related Quality of Life

Age-specific quality-of-life weights were derived from the Medical Expenditure Panel Survey [28], [29]. Quality-of-life reductions associated with chronic HCV infection were estimated by combining several studies [30], [31], [32], [33], [34]. We assumed an annual utility decrement from being aware of HCV infection [11], [35] and for being on treatment, varying these in sensitivity analyses [15], [31].

### Costs

Age-specific baseline healthcare costs included out-of-pocket expenses [36]. We included additional fibrosis stage-specific costs attributable to chronic HCV infection for patients unaware and aware of their infection status [37] which we reduced by 50% for patients who achieved sustained virologic response (SVR) [26], [37]. We estimated screening-related costs using the 2010 Medicare fee schedule. We included patients’ time costs during HCV diagnosis, liver biopsy, and IL-28B genotyping by multiplying time lost by the mean 2010 hourly wage [38]. Treatment costs include drugs and medical care for the duration of treatment, which depend on a patient’s virologic response to therapy [15]. Costs were inflation-adjusted to 2010 U.S. dollars using the Consumer Price Index [39].

### Cost-Effectiveness

Main outcomes were lifetime costs and quality-adjusted life-years (QALYs). Results are presented as population-weighted averages over race and sex [40]. We adopted a societal perspective, considered costs and benefits over a lifetime horizon, and discounted future costs and benefits at 3% annually [41]. We performed deterministic sensitivity analyses for all variables as well as probabilistic sensitivity analyses (Table S3 in Appendix S1).

## Results

### Main Analysis

The health benefits of both birth-cohort and risk-factor guided HCV screening compared to no screening are substantial but depend strongly on ensuring adequate treatment uptake and adherence.

**Figure 2:**
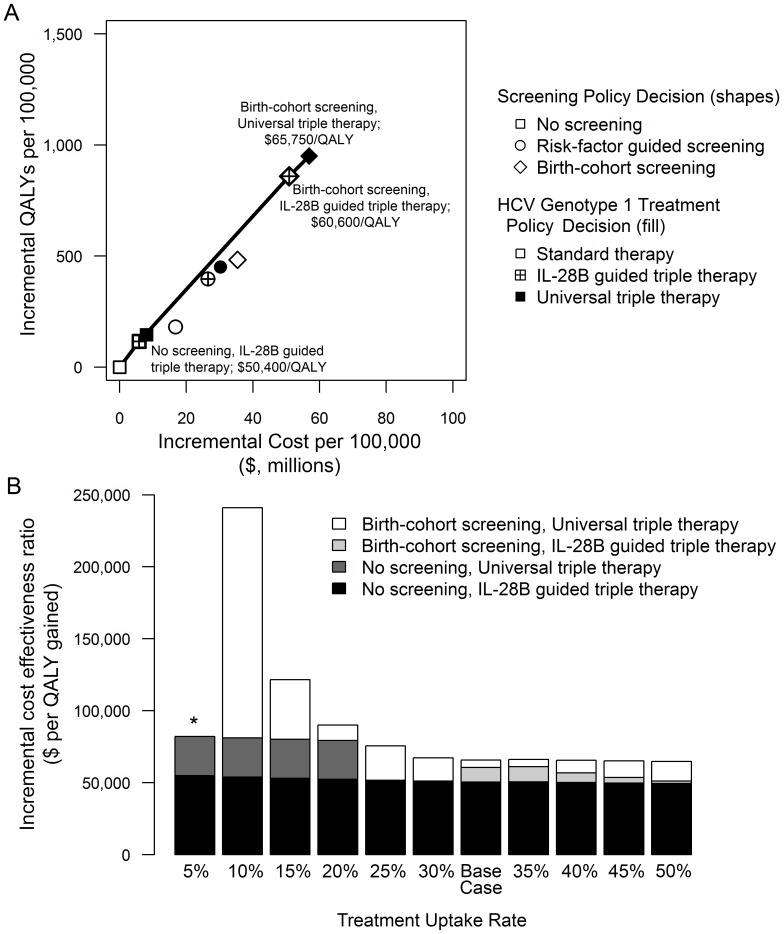
Cost-effectiveness analysis. (A) The graph plots the incremental discounted QALYs (*y*-axis) and incremental discounted lifetime costs (*x*-axis) for each combined screening and treatment strategy. The solid line represents the cost-effectiveness frontier, those strategies that are potentially economically efficient depending on one’s willingness-to-pay per unit of health benefit gained. (B) The bar graph shows the incremental cost-effectiveness ratios of each combined screening and treatment strategy at different levels of treatment uptake at each opportunity (varied over the range 0–50%). The asterisk denotes that, at 5% uptake, birth-cohort screening followed by universal triple therapy for screen-detected, treatment-eligible individuals is dominated. For both panels, IL-28B = interleukin-28B; QALY = quality-adjusted life-year.

In the base case, risk-factor guided and birth-cohort screening of individuals who are currently 50 years of age, respectively, averted 4–7 and 10–15 liver transplants, 13–27 and 35–56 liver cancers, and gained 181–450 and 483–950 QALYs per 100,000 people compared to no screening, depending on the HCV treatment strategy used and assuming 30–40% treatment uptake and 70% treatment adherence (Table 2). Risk-factor guided and birth-cohort screening, respectively, increased costs by $17–30 million and $35–57 million per 100,000 people compared to no screening. Birth-cohort screening yielded greater health benefits per dollar spent than risk-factor guided screening in all cases largely because risk factors for HCV are too common and not sufficiently predictive. Compared to no screening, birth-cohort screening of individuals who are currently 50 years of age followed by IL-28B-guided triple-therapy costs $60,590 per QALY gained. Birth-cohort screening followed by universal triple therapy costs $65,749 per QALY gained (Figure 2a). Costs and benefits of each screening and treatment strategy for cohorts of 40, 50, 60, and 70 year-olds are shown in Table 3. Birth-cohort screening followed by universal triple therapy costs less than $100,000 per QALY for ages 40–64 years compared to the next best strategy (Table 3). Section II in Appendix S1 presents sex and race-stratified results as well as results for settings in which IL-28B genotyping or triple therapy is not available (Table S4, Table S5 and Figure S2 in Appendix S1).

**Table 2:** Base case lifetime costs, health benefits (per 100,000), and incremental costs effectiveness ratio of combined screening and treatment strategies for a cohort of individuals who are currently 50 years of age.

COMBINED STRATEGIES		Per 100,000*				
Screening	Treatment	Liver Cancers Averted	Liver Transplants Averted	IncrementalCost ($)	IncrementalQALY	ICER($/QALY)
No Screening	Standard therapy	Reference	Reference	Reference	Reference	–
No Screening	IL-28B guided triple therapy	7	1	5,833,793	116	$50,417
No Screening	Universal triple therapy	9	2	8,076,805	145	Dominated
Risk-Based	Standard therapy	13	4	16,795,805	181	Dominated
Risk-Based	IL-28B guided triple therapy	24	6	26,537,268	397	Dominated
Risk-Based	Universal triple therapy	27	7	30,282,373	450	Dominated
Birth-cohort	Standard therapy	35	10	35,369,580	483	Dominated
Birth-cohort	IL-28B guided triple therapy	53	14	50,876,459	859	$60,590
Birth-cohort	Universal triple therapy	56	15	56,843,606	950	$65,749

* Population weighted average (white male 44%, white female 45%, black male 5%, black female 6%) for fibrosis distribution: F0 13%, F1 51%, F2 13%, F3 10%, and F4 13%. All incremental cost and QALY are compared to the reference.

ICER = incremental cost-effectiveness ratio; IL-28B = interleukin-28B; QALY = quality-adjusted life-year.

“Dominated” indicates that the strategy costs more and provides fewer benefits than another strategy or a combination of two strategies.

**Table 3:** Lifetime costs, health benefits (per 100,000), and incremental costs effectiveness ratio of combined screening and treatment strategies for various patient ages.

	COMBINED STRATEGIES		Per 100,000*		
Age	Screening	Treatment	IncrementalCost ($)	IncrementalQALY	ICER($/QALY)
**40**	No Screening	Standard therapy	Reference	Reference	–
	No Screening	IL-28B guided triple therapy	3,001,814	68	44,228
	No Screening	Universal triple therapy	4,151,745	84	Dominated
	Risk-Based	Standard therapy	8,740,935	66	Dominated
	Risk-Based	IL-28B guided triple therapy	13,124,293	176	Dominated
	Risk-Based	Universal triple therapy	14,811,803	202	Dominated
	Birth-cohort	Standard therapy	15,875,395	184	Dominated
	Birth-cohort	IL-28B guided triple therapy	22,457,464	366	Dominated
	Birth-cohort	Universal triple therapy	25,006,361	408	64,719
**50**	No Screening	Standard therapy	Reference	Reference	–
	No Screening	IL-28B guided triple therapy	5,833,793	116	50,417
	No Screening	Universal triple therapy	8,076,805	145	Dominated
	Risk-Based	Standard therapy	16,795,805	181	Dominated
	Risk-Based	IL-28B guided triple therapy	26,537,268	397	Dominated
	Risk-Based	Universal triple therapy	30,282,373	450	Dominated
	Birth-cohort	Standard therapy	35,369,580	483	Dominated
	Birth-cohort	IL-28B guided triple therapy	50,876,459	859	60,590
	Birth-cohort	Universal triple therapy	56,843,606	950	65,749
**60**	No Screening	Standard therapy	Reference	Reference	–
	No Screening	IL-28B guided triple therapy	4,454,805	62	71,897
	No Screening	Universal triple therapy	6,181,389	79	Dominated
	Risk-Based	Standard therapy	16,590,388	115	Dominated
	Risk-Based	IL-28B guided triple therapy	25,235,234	250	Dominated
	Risk-Based	Universal triple therapy	28,555,220	285	Dominated
	Birth-cohort	Standard therapy	35,040,741	317	Dominated
	Birth-cohort	IL-28B guided triple therapy	49,907,383	571	Dominated
	Birth-cohort	Universal triple therapy	55,601,271	636	89,074
**70**	No Screening	Standard therapy	Reference	Reference	–
	No Screening	IL-28B guided triple therapy	2,909,047	25	116,963
	No Screening	Universal triple therapy	4,058,590	32	153,204
	Risk-Based	Standard therapy	15,037,415	37	Dominated
	Risk-Based	IL-28B guided triple therapy	22,001,319	102	Dominated
	Risk-Based	Universal triple therapy	24,700,957	121	Dominated
	Birth-cohort	Standard therapy	31,250,082	112	Dominated
	Birth-cohort	IL-28B guided triple therapy	44,286,780	247	Dominated
	Birth-cohort	Universal triple therapy	49,318,701	285	179,186

* Population weighted average (white male 44%, white female 45%, black male 5%, black female 6%) for fibrosis distribution: F0 13%, F1 51%, F2 13%, F3 10%, and F4 13%. All incremental cost and QALY are compared to the reference.

ICER = incremental cost-effectiveness ratio; IL-28B = interleukin-28B; QALY = quality-adjusted life-year.

“Dominated” indicates that the strategy costs more and provides fewer benefits than another strategy or a combination of two strategies.

Lower levels of treatment uptake erode the cost-effectiveness of HCV screening (Figure 2b). If only 10% of the screened and treatment-eligible population initiate treatment at each opportunity, birth-cohort screening with triple therapy costs $241,100 per QALY compared to no screening. Birth-cohort screening costs approximately $50,000 per QALY only when treatment uptake is greater than 50%. The introduction of triple therapy into practice may improve treatment uptake due to its higher effectiveness, but its higher rates of side effects may also decrease adherence. If adherence to triple therapy is lower than standard therapy, the preferred strategy shifts from universal triple therapy to IL-28B genotype guided triple therapy (Table 4).

**Table 4:** Deterministic sensitivity analysis of cohort and treatment factors.

			ICER ($/QALY)*		
	No Screening,IL-28Bguided tripletherapy	No Screening,Universaltriple therapy	Birth-cohortscreening,Standard therapy	Birth-cohortscreening, IL-28Bguided tripletherapy	Birth-cohortscreening,Universaltriple therapy
**Cohort Characteristics (age and overall HCV prevalence)**					
40 years (2.82%**)	44,228	Dominated	Dominated	Dominated	64,719
50 years (2.98%)	50,530	Dominated	Dominated	62,329	65,870
50 years (4.27%, base case)	50,417	Dominated	Dominated	60,590	65,749
50 years (5.56%)	50,358	Dominated	Dominated	59,660	65,684
60 years (4.27%)	71,897	Dominated	Dominated	Dominated	89,074
**Initial Fibrosis Stage distribution**					
Less severe (30% F0, 41% F1, 22% F2,3% F3, and 4% F4)	54,222	Dominated	Dominated	71,337	73,031
More severe (18% F0, 24% F1, 17% F2,13% F3, and 28% F4)	46,939	Dominated	Dominated	53,746	59,246
**Quality of life reduction from awareness of HCV-positive status**					
No reduction	Dominated	Dominated	Dominated	48,863	70,173
High reduction (-0.05)	46,137	70,742	Dominated	Dominated	92,509
**Chronic HCV health care cost from awareness of HCV-positive status**					
High utilization (annual cost of $4,200in F0–F3, and $8,400 in F4)	36,944	66,682	Dominated	Dominated	100,167
Same cost and utility between aware andunaware of HCV-positive status (annual costof $1,400 in F0–F3, and $4,200 in F4)	Dominated	Dominated	28,279	44,092	70,173
**Treatment uptake**					
Very low uptake (10%)	53,938	81,115	Dominated	Dominated	241,066
Low uptake (20%)	52,370	79,357	Dominated	Dominated	90,129
Medium High uptake (50%)	49,447	Dominated	Dominated	51,165	64,786
High uptake (70%)	Dominated	Dominated	Dominated	45,306	63,602
Very high uptake (90%)	Dominated	Dominated	Dominated	42,160	62,866
**Treatment adherence** &					
Low adherence to triple therapy (50%)	Dominated	Dominated	73,265	79,538	Dominated
**Reduction of non-liver related mortality**					
No reduction	61,792	Dominated	Dominated	Dominated	83,980
High reduction	41,149	Dominated	Dominated	44,896	52,633

* Population weighted average (white male 44%, white female 45%, black male 5%, black female 6%). Each strategy is compared to the next-best strategy on the efficient frontier. Risk factors were considered for all of these scenario analyses but are dominated in all cases.

** Prevalence based on 1962–1971 cohort.

& Adherence is defined as patients taking ≥80% of their HCV medications.

“Dominated” indicates that the strategy costs more and provides fewer benefits than another strategy or a combination of two strategies.

### Population Impact

For the current screening eligible cohort of 85.3 million 40–64 year olds in the US, implementing a policy of risk-factor guided screening combined with IL-28B guided triple therapy increased 162,482 QALYs at an increased cost of $14.2 billion compared to no screening with IL-28B guided triple therapy; and combined with universal triple therapy increased 176,912 QALYs at an increased cost of $15.2 billion compared to no screening with universal triple therapy. Also compared to no screening with the same treatment strategy, implementing a policy of birth-cohort screening of 40–64 year olds combined with IL-28B guided triple therapy increased 436,394 QALYs at an increased cost of $30.1 billion; and combined with universal triple therapy increased QALYs by as much as 474,196 QALYs at an increased cost of $32.6 billion (Table 5).

**Table 5:** Population impact of HCV screening aged 40–64 years, total lifetime costs, health benefits, and incremental costs effectiveness ratio of combined screening and treatment strategies**.**

COMBINED STRATEGIES		Incremental Cost ($)	Incremental QALY	ICER ($/QALY)*
Screening	Treatment			
**No Screening**	Standard therapy	Reference	Reference	–
**No Screening**	IL-28B guided triple therapy	3,712,793,775	70,138	52,936
**No Screening**	Universal triple therapy	5,144,766,183	87,827	Dominated
**Risk-Based**	Standard therapy	11,635,472,594	102,088	Dominated
**Risk-Based**	IL-28B guided triple therapy	17,914,981,494	232,620	Dominated
**Risk-Based**	Universal triple therapy	20,332,866,318	264,739	Dominated
**Birth-cohort**	Standard therapy	23,673,786,131	277,782	Dominated
**Birth-cohort**	IL-28B guided triple therapy	33,801,361,880	506,532	68,948
**Birth-cohort**	Universal triple therapy	37,702,921,559	562,023	70,309

* Population weighted average (white male 44%, white female 45%, black male 5%, black female 6%) for fibrosis distribution: F0 13%, F1 51%, F2 13%, F3 10%, and F4 13%. All incremental cost and QALY are compared to the reference. Eligible screening population in the 40–64 year-old cohort is assumed at 83.5 million.

ICER = incremental cost-effectiveness ratio; IL-28B = interleukin-28B; QALY = quality-adjusted life-year.

“Dominated” indicates that the strategy costs more and provides fewer benefits than another strategy or a combination of two strategies.

### Screening in Different Birth Cohorts

**Figure 3:**
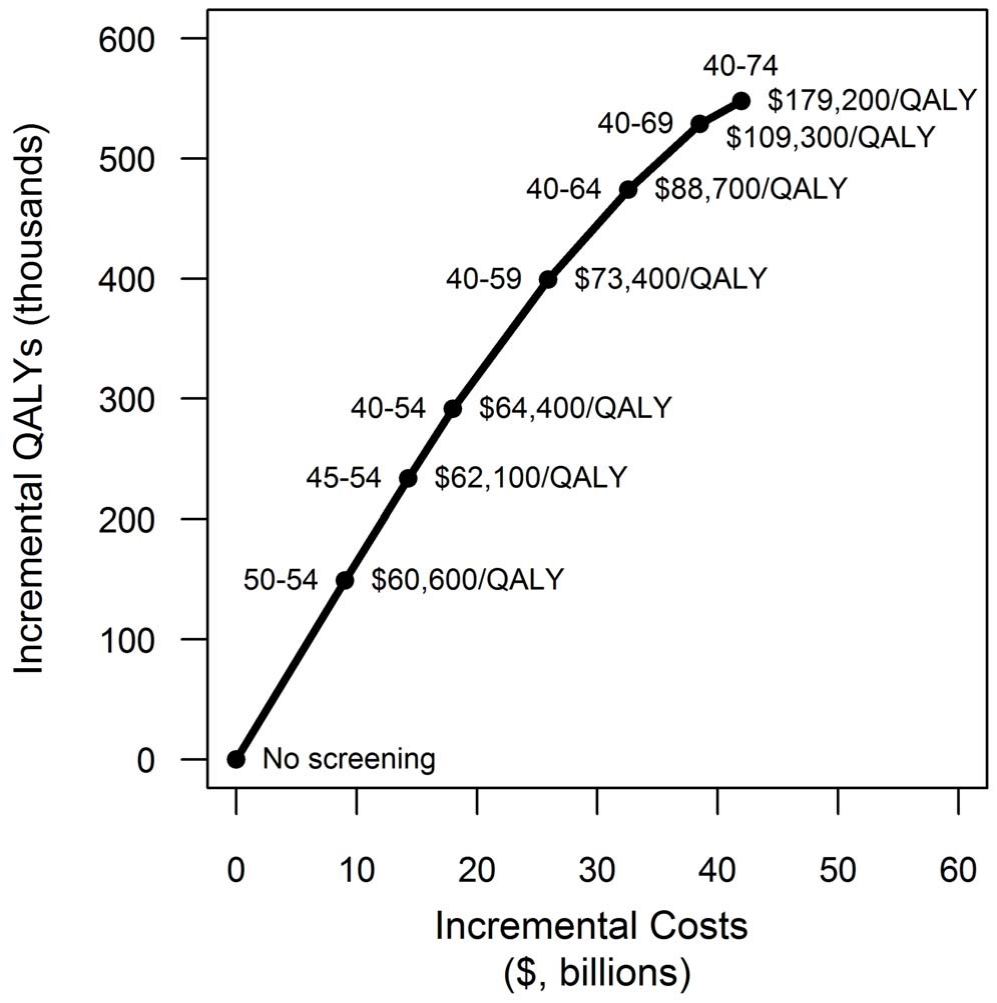
Cost-effectiveness of birth-cohort screening by age group. The graph plots the incremental discounted QALYs and incremental discounted lifetime costs for screening various birth cohorts. The analysis shown in the graph assumes that the treatment strategy used is universal triple therapy. For clarity, the graph shows only those strategies on the cost-effectiveness frontier (i.e., those that are not dominated) although all combinations of birth-cohort groups (40–44, 45–49, 50–54, 55–59, 60–64, 65–69, 70–74 years of age) were considered in the analysis.

Holding the treatment decision constant, we evaluated various possible birth-cohorts to include in an efficient screening program. In this analysis we are essentially evaluating the new CDC recommendation of one-time birth-cohort screening and answering the question: if it is not possible to screen all birth cohorts, which age groups should be screened to most effectively address chronic HCV infections in the US? If standard therapy continues to be the widely used treatment choice, screening individuals aged 40–59 costs $90,090 per QALY gained. Expanding screening to include the next older cohort, individuals aged 60–64 increases the cost to $110,576 per QALY gained. If IL-28B guided triple therapy or universal triple therapy became the widely adopted treatment actions, screening individuals aged 40–64 costs approximately $89,000 per QALY gained (Figure 3). Expanding screening to include the next older cohort, individuals aged 65–69, increases the cost to approximately $110,000 per QALY gained. Screening individuals aged 70–74 costs approximately $180,000 per QALY gained if either IL-28B guided or universal triple therapy are the treatment action and costs $277,800 per QALY gained if HCV-infected patients are treated with standard two-drug therapy.

### Sensitivity Analyses

#### Deterministic sensitivity analyses

The impact of varying cohort age and HCV prevalence, screening-related factors, and treatment-related factors on the findings are presented in Table 4 (with additional information provided in Section II, Table S6 in Appendix S1).

Our main analysis considered individuals aged 50 in 2011. Birth-cohort screening followed by universal triple therapy cost $64,700, $65,700, $89,100, and $179,200 per QALY gained for 40 year-olds, 50 year-olds, 60 year-olds, and 70 year-olds respectively. In the oldest cohorts, high HCV prevalence is offset by the shorter horizon over which to accrue benefits from averted serious liver diseases.

Screening factors such as the ability to elicit a true risk history from patients, the cost of risk assessment, the test characteristics, and costs of HCV screening and diagnostic tests did not alter the main results.

The fibrosis-stage distribution of individuals diagnosed through screening strongly influences cost-effectiveness (Figure S1 in Appendix S1). If more HCV-infected individuals detected via screening have mild liver fibrosis, screening is less cost-effective. If the fibrosis distribution were 71% F0–F1 and 29% F2–F4 [12], birth-cohort screening with triple therapy costs $73,000 per QALY whereas if the distribution were 42% F0–F1 and 58% F2–F4 [42], it costs $59,200 per QALY.

People newly diagnosed with HCV may increase their use of health care services both to manage their illness and because they become concerned, anxious, or worried, also leading to a lower quality-of-life. If this occurs, birth-cohort screening 50 year olds costs over $100,200 per QALY (Table 4). Proper counseling may help to ensure that patients do not experience decreased quality-of-life or worry-related increases in heath-resource consumption.

While chronic HCV treatment may also reduce non-liver related mortality [22], some studies suggest that this is because patients selected for treatment are relatively healthier. If non-liver related mortality does not change with successful HCV treatment, birth-cohort screening followed by triple therapy costs $84,000 per QALY.

Interferon-free direct-acting antiviral drugs that are currently in phase I and phase II trials show potential for high efficacy and reduced side-effects. As of yet, these therapies have not established efficacy and safety profiles in large phase III trials nor have they been FDA approved for use in routine care. We included a scenario analysis in which treatment is more effective (95% SVR for HCV genotype 1 patients with IL-28B genotype of CC and 80% SVR for HCV genotype 1 patients with non-CC IL-28B genotypes), with a better side-effect profile (QALY decrements of 0.025 instead of 0.055), and cost 70% of triple therapy (due to lower drug costs and/or shorter therapy duration). Result showed birth-cohort screening followed by universal triple therapy was preferred for birth cohorts of ages 40–64 years with a cost between $30,000 and $50,000 per QALY gained.

#### Probabilistic sensitivity analyses

At a willingness-to-pay threshold of $50,000 per QALY, screening was optimal 25% of the time. However, at a willingness-to-pay of $60,000 and $100,000 per QALY, birth-cohort screening was optimal 60% and 98% of the time, respectively. If treatment uptake were only 20%, even at $100,000 per QALY, birth-cohort screening was optimal only 67% of the time. Risk-factor guided screening was never preferred to birth-cohort screening (Figure S3 in Appendix S1).

## Discussion

An estimated 2 million Americans are unaware of their chronic HCV infections. The health and mortality burdens experienced by this group are expected to rise as many were infected more than 30 years ago and have experienced long-term, asymptomatic liver fibrosis progression. Because newer treatments are more effective, early disease identification and treatment via birth-cohort screening of individuals who are currently 40 to 64 years old could improve health and increase life expectancy. A dramatic expansion of the current screening and treatment programs would also be costly. If high treatment uptake rates can be maintained for screen-detected individuals, compared to no systematic screening, birth-cohort screening costs between $60,590 and $65,749 per QALY gained depending on the recommended treatment regimen. However, if treatment uptake were lower, birth-cohort screening could cost over $200,000 per QALY gained.

Our study expands upon results from prior cost-effectiveness analyses of U.S. HCV screening policies [12], [13], [14]: explicitly examining risk-factor guided screening via detailed modeling of risk assessment; incorporating mortality hazard rates stratified by risk group and HCV status which permit more accurate estimates of the potential benefits of HCV screening and treatment; considering the role of IL-28B-guided triple therapy; and considering the cost-effectiveness of screening in age groups of 40–74 years of age. Like other recent studies, we find that birth-cohort screening is more cost-effective than risk-based screening for 40–64 year-olds. However, unlike these studies, the cost per QALY gained that we estimate is substantially higher, especially in scenarios in which treatment uptake is lower, the costs of downstream chronic HCV care are higher, or the quality of chronic HCV care worsens due to capacity limitations with large numbers of new screen-detected individuals.

We closely examined risk-factor guided HCV screening and found that it is not preferred to birth-cohort screening for several reasons. First, in the case of HCV, NHANES-derived prevalence estimates show that a relatively large fraction of infected individuals (28–47%) have no known risk factors. Second, risk factors such as history of injection drug use, blood transfusion before 1992, and greater than 20 lifetime sex partners are also relatively common among uninfected individuals and, therefore, are not sufficiently predictive. Third, chronically infected individuals without risk factors are often better treatment candidates than individuals with risk factors due to lower overall mortality from comorbidities. Finally, identifying patients with stigmatized risk factors, such as a history of injection drug, use may prove difficult. Despite these challenges, risk-based screening may still be important given tightening healthcare budgets. Implementing birth-cohort screening of individuals currently age 40–64 years costs between $12.0 and $17.4 billion more than implementing risk-based screening.

Maintaining high levels of treatment uptake and adherence is crucial for ensuring the cost-effectiveness of HCV screening. It is possible that treatment initiation rates will increase with the introduction of more effective triple therapy or direct-acting antiviral-only regimens when available, but these drugs’ side effects may also decrease adherence. In parts of the U.S., large numbers of newly screen-detected treatment candidates may exceed existing capacity, effectively lowering uptake rates.

The importance of providing appropriate counseling to reduce HCV related co-morbidities and minimize quality-of-life loss from awareness of HCV-positive status as part of HCV screening and treatment cannot be overstated. A driver of the relatively high cost per QALY gained from HCV screening is that knowing one’s HCV status reduces quality-of-life given anxiety surrounding the daunting possibility of intense treatment along with potential side effects and social stigma [11], [35]. If proper counseling can mitigate these effects and reduce associated increases in health resource consumption, birth-cohort screening of 50-year olds with IL-28B guided triple therapy costs $44,100 per QALY compared to no screening.

Our study has several limitations. First, we analyzed NHANES to estimate the prevalence of HCV stratified by sex, race, and risk-factor status depending on relatively small sample sizes. Reassuringly, varying these parameters across the range of uncertainty does not alter our main findings. Second, we did not include people with HIV or hepatitis B co-infections due to complexities of the two diseases and clinical challenges of safely and effectively treating both diseases [43], [44]. Third, observational studies that provide long-term follow-up data on differential mortality rates among people in various risk groups and HCV infection statuses are limited. We used the NHANES III linked mortality data, but there remains uncertainty about this effect. Fourth, because the protease inhibitors used in triple therapy were only recently approved, the feasibility of implementing response-guided therapy in routine practice, treatment effectiveness, and adherence are unknown. Finally, we did not include reductions in HCV transmission due to screening and successful treatment and, thus, may underestimate benefits associated with screening. Transmission effects are likely limited because our targeted screening group, older American adults, is responsible for a low percentage of HCV transmission [45].

One-time, birth-cohort HCV screening at a routine medical visit for asymptomatic adults currently aged 40–64 years followed by IL28B-guided or universal triple therapy for HCV infected patients provides substantial benefits and is likely cost-effective provided a sufficiently high treatment uptake rate and quality of follow-on care are ensured. Along with providing birth-cohort screening, HCV policies should focus on ensuring that screen-detected individuals receive prompt treatment and high-quality HCV care.

## Supporting Information

Appendix S1 Contains appendices with supporting information.
(DOCX)
