# Supplementary material for: Cost-Effectiveness Analysis of Risk-Factor Guided and Birth-Cohort Screening for Chronic Hepatitis C Infection in the United States
Source: PLoS One. 2013 Mar 22;8(3):e58975. doi: 10.1371/journal.pone.0058975 (PMC3606430; doi:10.1371/journal.pone.0058975)
Supplement: Appendix S1 — Contains appendices with supporting information. (DOCX) [file pone.0058975.s001.docx]

**Supporting Information**

**Cost-Effectiveness Analysis of Risk-Factor Guided and Birth-cohort Screening for Chronic Hepatitis C Infection in the United States**

Shan Liu, SM^1^; Lauren E. Cipriano, MS^1^; Mark Holodniy, MD^2^; and Jeremy D. Goldhaber-Fiebert, PhD^3^

(1) Department of Management Science and Engineering, Stanford University, Stanford, CA, USA

(2) VA Palo Alto Health Care System, Palo Alto, CA, USA

(3) Stanford Health Policy, Centers for Health Policy and Primary Care and Outcomes Research, Stanford University, Stanford CA, USA

**Appendix S1**

**I: Supplemental Information on Data and Methods**

**Model Implementation**

We implemented the model in TreeAge Pro 2009 Suite (TreeAge Software, Williamstown, Massachusetts) and Microsoft Excel 2007 (Microsoft Corp., Redmond, Washington).

**Statistical Analyses on HCV Prevalence and Risk Factors**

We estimated the prevalence of risk factors and the prevalence of HCV among high- and low-risk individuals stratified by age, sex and race using data from the National Health and Nutrition Examination Survey (NHANES) (2001-2008). We defined a high-risk person as someone having a history of injection drug use, transfusion prior to 1992, or greater than 20 lifetime sex partners. We combined all survey years to estimate prevalence for the 1952-1961 birth cohort (those aged 50-59 years old currently) in the base case, using the 1962-1971 birth cohort prevalence (those age 40-49 years old currently) in sensitivity analyses (*n* = 5,654). We used logistic regression to predict the prevalence of being high-risk based on sex, race, and age accounting for sample weighting and NHANES complex sampling design. Similarly, we used logistic regression to predict the prevalence of individuals with HCV antibodies using sex, race, risk status, and age.

**Statistical Analyses on Mortality from Non-HCV causes**

No single study contains data needed to estimate subgroup-specific prevalence of HCV, risk factor status, and mortality risks. We developed a combined modeling approach to infer necessary risk-group-specific mortality rates for chronically HCV-infected U.S. adults.

We analyzed the NHANES III–linked mortality data in which HCV status was assessed from 1988 to 1994 with mortality follow-up of the same persons through 2006 (*n* = 15,892). We constructed Cox proportional hazard models to estimate the mortality hazard ratios for all-cause death by sex, race, HCV, risk status (greater than 20 lifetime sex partners), interaction between HCV and risk status, age, and age-squared variables for people between ages 17-60, excluding cases with missing risk information. Controlling for age, we used four hazard ratios (male, black, HCV positive, and high risk) to calculate the 16 mortality hazard ratios shown in Table S1. We adjusted the hazard ratio for HCV infection down using a factor of 0.8 since it is estimated that for HCV-infected individuals, 20% of mortality is liver-related, which is accounted separately in the HCV natural history model.

Using the 16 estimated hazard ratios, we calculated the population-weighted average mortality to match the 2006 U.S. life-table over age 50-100, based on the prevalence of HCV and risk status by sex and race from NHANES (2001-2008) data analyses and the U.S. 2009 census distribution for people aged 50-54 (non-black male 44%, non-black female 45%, black male 5%, and black female 6%). To avoid overestimation of death in the older ages, we linearly attenuated the 16 hazard ratios starting from age 70 down to 1.0 by age 100. We adjusted the mortality rates for people who are recovered from HCV by a linear combination of their mortality rates with HCV and mortality rates without HCV using a factor of 0.7.

**Table S1. Mortality hazard ratios**

|  |  | Overall (95% CI) |  |  |
| --- | --- | --- | --- | --- |
|  | Male | 1.32 (1.05-1.66) |  |  |
|  | Black | 1.74 (1.45-2.10) |  |  |
|  | HCV positive | 3.46 (2.00-5.97) |  |  |
|  | High risk (>=20 sex partners) | 1.41 (1.03-1.91) |  |  |
| Males | **White** |  | **Black** |  |
|  | <20 Sex Partners | >=20 Sex Partners | <20 Sex Partners | >=20 Sex Partners |
| No HCV | 1.32 | 1.85 | 2.29 | 3.23 |
| HCV | 3.64 | 5.12 | 6.34 | 8.92 |
| Females | **White** |  | **Black** |  |
|  | <20 Sex Partners | >=20 Sex Partners | <20 Sex Partners | >=20 Sex Partners |
| No HCV | 1 | 1.41 | 1.74 | 2.45 |
| HCV | 2.76 | 3.89 | 4.81 | 6.77 |
|  |  |  |  |  |

**HCV Response-Guided Therapy**

We evaluated the cost-effectiveness of alternative HCV response-guided therapies in a previous paper [1]. We use the models of each treatment from this prior analysis, briefly summarizing the details here.

*Standard Therapy*

Standard therapy includes pegylated interferon alfa (2a or 2b) and ribavirin for 48 weeks. Virologic response is assessed at 12, 24, and 48 weeks. Patients who do not achieve an early virologic response (EVR) (defined as a ≥2-log_10_/mL reduction or complete absence of serum HCV RNA at week 12 of treatment compared with the baseline level), discontinue treatment and are considered nonresponsive. Patients with detectable serum HCV RNA at 24 weeks discontinue treatment and are considered partially responsive; otherwise, patients continue treatment for a total of 48 weeks. Patients who do not achieve SVR at the end of treatment are considered to have relapsed.

*Triple Therapy*

The characteristics of triple therapy are estimated on the basis of both HCV drugs recently approved by the U.S. Food and Drug Administration, telaprevir and boceprevir. Given the differences in study populations and the broad overlaps in their effectiveness estimates, we considered a single protease inhibitor. In the base-case analysis, similar to boceprevir, we assumed that patients receive a course of triple therapy during the first 28 weeks (4 weeks of pegylated interferon and ribavirin lead-in followed by 24 weeks of triple therapy). Patients who do not achieve EVR at week 12 of treatment discontinue therapy and are considered nonresponsive. At week 24, treatment is again discontinued in some patients because of nonresponse. However, among patients who respond to treatment, a proportion will stop treatment early because they meet completion criteria at week 28. The remaining patients continue an 8 additional weeks of triple therapy followed by pegylated interferon and ribavirin alone until 48 weeks. Treatment effectiveness is presented in Table S2. Additional cost-effectiveness results using triple therapy with telaprevir can be found in Table S6.

**Table S2. Treatment effectiveness for genotype 1 patients [1]**

| Effectiveness of treatment in genotype 1 patients  (CC/non-CC) | Base Case (Range) | Reference |
| --- | --- | --- |
| Standard therapy (PEG-INF+Rb) |  | [2,3,4] |
| Mild fibrosis (F0/F1/F2), white |  |  |
| Probability of EVR (assessed at 12 wk) | 0.90/0.66 |  |
| Probability of virologic response at 24 wk,  conditional on EVR | 0.92/0.75 |  |
| Probability of SVR, conditional on completed  treatment (48 wk) | 0.83/0.64 |  |
| Overall probability of SVR† | 0.46 (0.42–0.49) |  |
| Mild fibrosis (F0/F1/F2), black |  |  |
| Probability of EVR (assessed at 12 wk) | 0.76/0.45 |  |
| Probability of virologic response at 24 wk,  conditional on EVR | 0.95/0.78 |  |
| Probability of SVR, conditional on completed  treatment (48 wk) | 0.67/0.40 |  |
| Overall probability of SVR† | 0.19 (0.13–0.24) |  |
|  |  |  |
| Triple therapy (PEG-INF+Rb+PI)‡ |  | [5,6,7,8,9,10] |
| Adherence to triple therapy | 0.70 (0.50–0.70) |  |
| Mild fibrosis (F0/F1/F2), white |  |  |
| Probability of EVR (assessed at 12 wk) | 0.98/0.90 |  |
| Probability of treatment failure at 24 wk | 0.10/0.15 |  |
| Probability of treatment completion at either 24 or  28 wk | 0.62/0.43 |  |
| Probability of continuing treatment until 48 wk | 0.28/0.42 |  |
| Probability of SVR, conditional on completed  treatment (24 or 28 wk) | 0.98/0.95 |  |
| Probability of SVR, conditional on completed  treatment (48 wk) | 0.75/0.65 |  |
| Overall probability of SVR† | 0.68 (0.60–0.72) |  |
| Mild fibrosis (F0/F1/F2), black |  |  |
| Probability of EVR (assessed at 12 wk) | 0.80/0.60 |  |
| Probability of treatment failure at 24 wk | 0.14/0.14 |  |
| Probability of treatment completion at either 24 or  28 wk | 0.48/0.48 |  |
| Probability of continuing treatment until 48 wk | 0.38/0.38 |  |
| Probability of SVR, conditional on completed  treatment (24 or 28 wk) | 0.95/0.89 |  |
| Probability of SVR, conditional on completed  treatment (48 wks) | 0.70/0.60 |  |
| Overall probability of SVR† | 0.42 (0.24–0.47) |  |
| Reduction in SVR for advanced fibrosis stage (F3 and  F4) | 0.80 (0.70–1.00) |  |

EVR = early virologic response; HCC = hepatocellular carcinoma; HCV = hepatitis C virus; IL-28B = interleukin-28B; NHANES III = Third National Health and Nutrition Examination Survey; PEG-IFN = pegylated interferon; PI = protease inhibitor; Rb = ribavirin; SVR = sustained virologic response.

† Calculated final SVR for the full cohort stratified by race, but not by IL-28B genotypes.

‡ The reported triple therapy effectiveness used in the base-case analysis is similar to boceprevir.

**Probabilistic Sensitivity Analyses**

In probabilistic sensitivity analyses, model parameters were sampled simultaneously 2,000 times from their respective uncertainty distributions for groups defined in terms of race and sex. We used a normal, gamma, or beta distribution to capture the mean and range of each model variable reported in literature (Table S3). For each strategy in each simulation, we calculated the net monetary benefit ([total QALYs × willingness-to-pay] − total cost). We then constructed cost-effectiveness acceptability curves showing the probability that a given strategy yields the highest net monetary benefit at various willingness-to-pay thresholds.

**Table S3. Probabilistic sensitivity analysis parameter distribution assumptions**

| Variable | Distribution^*^ | Parameters |
| --- | --- | --- |
| Cohort characteristics |  |  |
| Proportion with HCV genotype 1 | Normal | mean, 0.800 (SD, 0.025) |
| Proportion with IL-28B genotype, CC-type polymorphism (vs. non–CC type) |  |  |
| White | Beta | a = 618; b = 1,053 |
| Black | Beta | a = 234; b = 1,437 |
|  |  |  |
| Risk Status prevalence (by sex, race)^&^ | Table of correlated prevalence |  |
| HCV prevalence (by sex, race, risk status)^&^ | Table of correlated prevalence |  |
|  |  |  |
| Screening characteristics |  |  |
| Probability of identified as high risk among true high risk individuals (sensitivity) | Normal | mean, 0.60 (SD, 0.05) |
| Annual rate of chance identification of HCV+ | Normal | mean, 0.037 (SD, 0.006) |
|  |  |  |
|  |  |  |
| HCV natural history |  |  |
| Proportion of patients with no fibrosis (F0) who do not progress | Normal | mean, 0.24 (SD, 0.02) |
| Annual probability of spontaneous remission from no fibrosis (F0) health state | Normal | mean, 1.19% (SD, 0.25%) |
| Fibrosis progression (annual probability) |  |  |
| Males |  |  |
| Age 40–49 y | Normal | mean, 5.26% (SD, 1.34%) |
| Age 50–59 y | Normal | mean, 11.75% (SD, 2.57%) |
| Age 60–69 y | Normal | mean, 19.83% (SD, 4.69%) |
| Age ≥70 y | Normal | mean, 25.99% (SD, 7.18%) |
| Females |  |  |
| Age 40–49 y | Normal | mean, 2.76% (SD, 0.75%) |
| Age 50–59 y | Normal | mean, 6.29% (SD, 1.83%) |
| Age 60–69 y | Normal | mean, 10.77% (SD, 3.54%) |
| Age 70–79 y | Normal | mean, 14.27% (SD, 3.92%) |
| Age ≥80 y | Normal | mean, 18.94% (SD, 6.29%) |
| Cirrhosis to decompensated cirrhosis | Normal | mean, 3.92% (SD, 0.40%) |
| Cirrhosis (both F4 and decompensated cirrhosis) to HCC | Normal | mean, 2.08% (SD, 0.20%) |
| Liver transplant (annual probability) |  |  |
| Decompensated cirrhosis to liver transplant | Gamma | α = 0.10; λ = 1/54,232 |
| HCC to liver transplant | Gamma | α = 1.47; λ = 1/11,031 |
| Chronic HCV conversion factor |  |  |
| Male | Beta | a = 880; b = 347 |
| Female | Beta | a = 796; b = 431 |
| Reduction factor on background mortality after successful treatment | Beta | a = 258; b = 110 |
| Liver-related mortality (annual probability) |  |  |
| Liver transplant | Normal | mean, 14.10% (SD, 0.40%) |
| After liver transplant | Normal | mean, 4.99% (SD, 0.06%) |
| Decompensated cirrhosis | Normal | mean, 26.36% (SD, 4.40%) |
| HCC |  |  |
| First year | Normal | mean, 72.00% (SD, 5.63%) |
| Subsequent year | Normal | mean, 25.31% (SD, 3.34%) |
| Treatment-related mortality | Normal | mean, 0.45% (SD, 0.20%) |
| Liver biopsy-related mortality | Normal | mean, 0.03% (SD, 0.01%) |
| Probability of FibroTest showing F2+ for patients in F0-F1 fibrosis stage | Beta | a = 141; b = 942 |
|  |  |  |
| Treatment characteristics |  |  |
| Percent of treatment eligible among diagnosed HCV+ | Beta | a = 59 ; b = 374 |
| Effectiveness of treatment in genotype 1 patients^**^ | Table of correlated effectiveness |  |
| Effectiveness of treatment in genotype 2&3 patients | Beta | a = 358; b = 90 |
| Reduction in SVR for advanced fibrosis stage (F3 and F4) | Normal | mean, 0.80 (SD, 0.05) |
|  |  |  |
| Quality of life† |  |  |
| HCV mild fibrosis (F0, F1) | Beta | a = 5.88; b = 0.12 |
| SVR after mild fibrosis | Beta | a = 5.88; b = 0.12 |
| HCV moderate fibrosis (F2, F3) | Beta | a = 38; b = 7 |
| SVR after moderate fibrosis | Beta | a = 34; b = 2 |
| Compensated cirrhosis (F4) | Beta | a = 40; b = 11 |
| SVR after cirrhosis | Beta | a = 34; b = 2 |
| Decompensated cirrhosis | Beta | a = 36; b = 14 |
| HCC | Beta | a = 36; b = 14 |
| Liver transplant (during or after) | Beta | a = 8; b = 2 |
| Standard therapy annualized decrement | Normal | mean, –0.11 (SD, 0.045) |
| Triple therapy annualized decrement | Normal | mean, –0.05 (SD, 0.03) |
| Liver transplant annualized decrement | Normal | mean, –0.20 (SD, 0.08) |
| Liver biopsy decrement | Normal | mean, –0.05 (SD, 0.01) |
| HCV awareness annualized decrement | Normal | mean, –0.02 (SD, 0.01) |
|  |  |  |
| Cost (2010 U.S. dollars), *$* |  |  |
| Screening |  |  |
| HCV anti-body screening (ELISA) | Normal | mean, 20 (SD, 5) |
| Risk identification (HCV+) | Normal | mean, 36 (SD, 9) |
| Diagnosis (ELISA to confirm, RIBA, and RNA test) | Normal | mean, 210 (SD, 52) |
| Reporting to the patient the results of a negative test | Normal | mean, 8 (SD, 2) |
| HCV genotyping | Normal | mean, 369 (SD, 92) |
| IL-28B genotyping | Normal | mean, 371 (SD, 93) |
| Liver biopsy | Normal | mean, 1,340 (SD, 155) |
| FibroTest | Normal | mean, 240 (SD, 30) |
| Treatment (drug and medical care) |  |  |
| PEG-INF+Rb (48 wk) | Normal | mean, 35 416 (SD, 3,500) |
| PIs (per week) | Normal | mean, 1,100 (SD, 56) |
| AEs, standard therapy | Normal | mean, 1,920 (SD, 288) |
| AEs, standard therapy, PI | Normal | mean, 2,586 (SD, 388) |
| Annual care\|\| |  |  |
| Aware of HCV status |  |  |
| HCV mild fibrosis (F0, F1) | Normal | mean, 1,410 (SD, 141) |
| HCV portal fibrosis (F2) | Normal | mean, 1,410 (SD, 141) |
| HCV bridging fibrosis (F3) | Normal | mean, 1,410 (SD, 141) |
| Compensated cirrhosis (F4) | Normal | mean, 4,194 (SD, 210) |
| Unaware of HCV status^‡^ |  |  |
| Decompensated cirrhosis | Normal | mean, 11,109 (SD, 2,780) |
| HCC | Normal | mean, 44,224 (SD, 11,054) |
| Liver transplant, first year | Normal | mean, 145,640 (SD, 36,410) |
| Liver transplant, subsequent | Normal | mean, 25,430 (SD, 6,358) |
| Recovered states from F0 to F4 (reduction factor from cost prior to treatment) | Normal | mean, 0.50 (SD, 0.05) |

AE = adverse event; EVR = early virologic response; HCC = hepatocellular carcinoma; HCV = hepatitis C virus; IL-28B = interleukin-28B; PEG-INF = pegylated interferon; PI = protease inhibitor; Rb = ribavirin; SD = standard deviation; SVR = sustained virologic response.

*We used Beta, Gamma, and Normal distributions to describe the uncertainty around model parameters. Distributions are parameterized as follows: Beta(a, b) where the mean of the distribution is a/[a+b] and the variance is [a × b]/[(a+b+1) × (a+b)^2^ ] ; Gamma(α, λ) where the mean of the distribution is α/ λ and the variance is α/ [λ^2^]; and Normal which is directly parameterized using its mean and standard deviation and is truncated if the parameter has an upper or lower limit.

& The table of correlated prevalence of high-risk individuals contains 10,000 rows and four columns; each row contains the proportion of high risk individuals across gender and race. The table of correlated HCV prevalence given gender, race, and risk status contains 10,000 rows and eight columns; each row contains the prevalence of HCV across gender, race and risk status. At each simulation, a random row is selected to be used as the prevalence for that simulation.

**The table of correlated effectiveness contains 100 rows; each row contains a complete set of virologic response profile for the entire duration of treatment stratified by race and IL-28B genotype. At each simulation, a random row is selected to be used as the treatment effectiveness profile for that simulation [1].

† Patients’ utilities for chronic HCV states and recovery states have a preference ranking order in the model. The utility of: mild chronic HCV ≥ moderate chronic HCV ≥ F4 ≥ decompensated cirrhosis, HCC; SVR after mild HCV ≥ mild chronic HCV; SVR after moderate HCV ≥ moderate chronic HCV; SVR after cirrhosis ≥ F4; after liver transplant ≥ decompensated cirrhosis, HCC; SVR after mild HCV ≥ SVR after moderate HCV ≥ SVR after cirrhosis.

||Patients’ annual care cost for chronic HCV states have a ranking order in the model. The cost of: mild chronic HCV (F0, F1) ≤ F2 ≤ F3≤ F4

‡The cost in the unaware F0-F3 states is assumed to be 60% of the cost in the aware F0-F3 states. The cost in the unaware F4 state is assumed to be 40% of the cost in the aware F4 states. These proportions are consistent with the base case differences.**II: Supplemental Results**

**Scenario Analysis on Fibrosis Stage**

Since chronic HCV is a slowly progressing disease whose health effects may not be experienced prior to death from other causes, screening in populations whose distribution of liver fibrosis tends to be milder costs more per QALY gained. For example, if all asymptomatic individuals infected with chronic HCV had F0 fibrosis, screening cost >$195,000 per QALY whereas if all had F4 fibrosis, screening cost $41,300 per QALY. Population-representative data on fibrosis distributions in U.S. individuals with chronic HCV who have not been diagnosed is difficult to obtain and hence the fibrosis distribution in this group is highly uncertain, a priority for additional research given its influence on the preferred screening policy (Figure S1).

**Figure S1. Cost effectiveness results by fibrosis stage of the cohort. The incremental cost effectiveness ratio of strategies on the efficient frontier compared to the next best strategy on the frontier.**


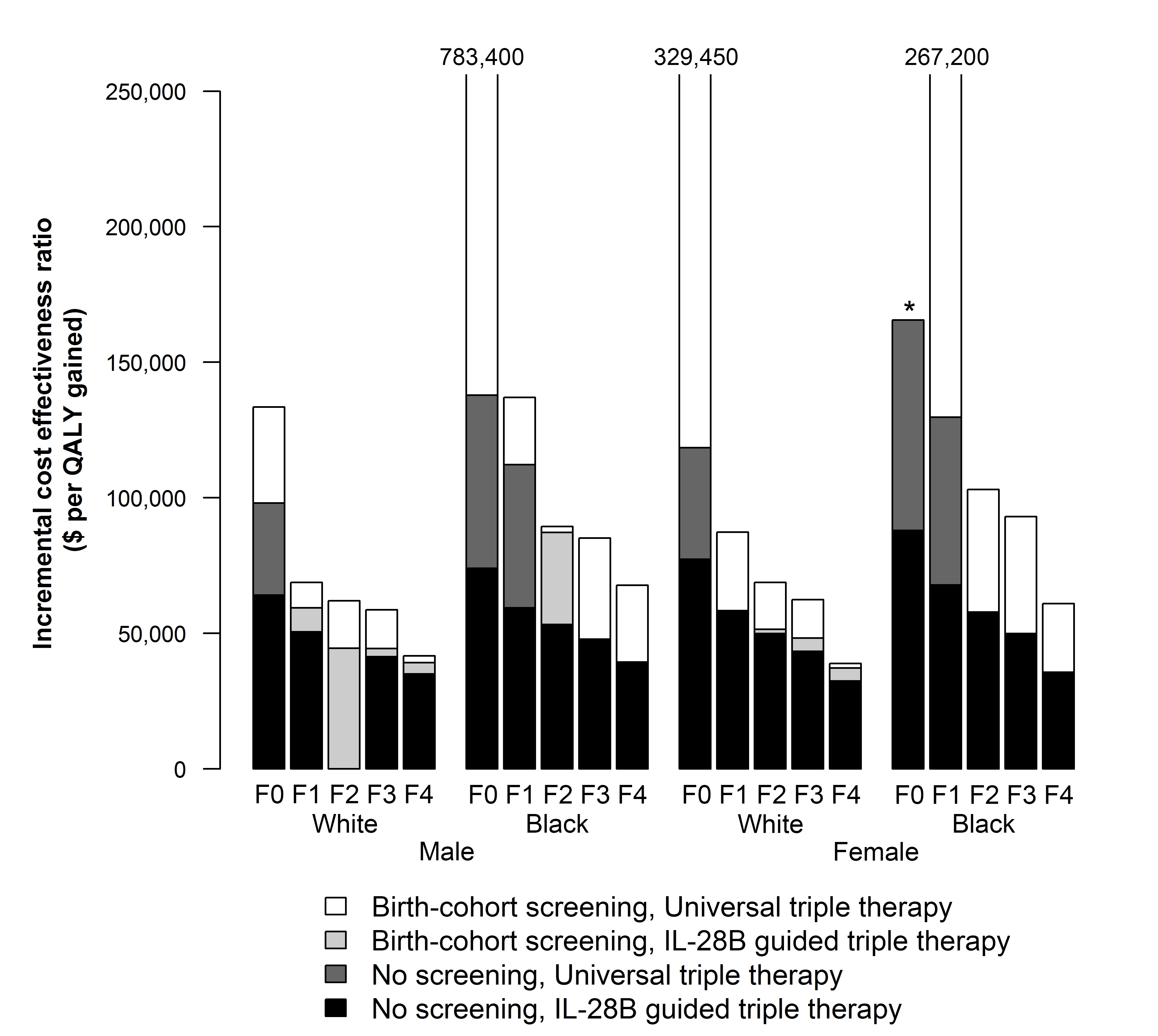


**Additional cost-effectiveness results by sex and race**

Combined differences in HCV prevalence, rates of fibrosis progression, IL-28B genotype distribution, treatment effectiveness, and background mortality influence the cost-effectiveness of the screening and treatment strategies considered. As these differences are correlated with race and sex, we present cost-effectiveness findings in these groups below (Table S4 and Figure S2). In general, lower HCV prevalence, slower rates of fibrosis progression and higher rates of background mortality reduce benefits and increase costs derived from screening most substantially. Similarly, IL-28B genotypes and differential treatment effectiveness as well as differential background mortality play important roles in the benefits and costs produced by treatment.

**Table S4. Base case lifetime costs, health benefits (per 100,000), and incremental costs effectiveness ratio of comprehensive screening and treatment strategies, by race and sex^*^.**

| **STRATEGY** | | Per 100,000 | | | | |
| --- | --- | --- | --- | --- | --- | --- |
| Screening Strategy | Treatment Strategy | Liver Cancers Averted | Liver Transplants Averted | Incremental Cost ($) | Incremental QALY | ICER ($/QALY) |
| **White Men** | |  |  |  |  |  |
| No Screening | Standard therapy | Reference | Reference | Reference | Reference | – |
| No Screening | IL-28B guided triple therapy | 9.5 | 2.1 | 6,974,881 | 146 | $47,827 |
| No Screening | Universal triple therapy | 11.8 | 2.6 | 9,938,640 | 186 | Dominated |
| Risk-Based | Standard therapy | 19.4 | 5.6 | 20,331,475 | 276 | Dominated |
| Risk-Based | IL-28B guided triple therapy | 34.5 | 9.3 | 32,122,717 | 551 | Dominated |
| Risk-Based | Universal triple therapy | 38.1 | 10.2 | 37,174,040 | 627 | Dominated |
| Birth-cohort | Standard therapy | 50.8 | 14.5 | 44,027,111 | 724 | Dominated |
| Birth-cohort | IL-28B guided triple therapy | 74.8 | 20.7 | 62,914,917 | 1,205 | $52,806 |
| Birth-cohort | Universal triple therapy | 80.6 | 22.3 | 71,024,181 | 1,334 | $62,875 |
| **White Women** | |  |  |  |  |  |
| No Screening | Standard therapy | Reference | Reference | Reference | Reference | – |
| No Screening | IL-28B guided triple therapy | 4.8 | 0.8 | 4,319,246 | 83 | $52,304 |
| No Screening | Universal triple therapy | 6.0 | 1.0 | 6,149,241 | 105 | Dominated |
| Risk-Based | Standard therapy | 8.0 | 2.1 | 12,450,345 | 120 | Dominated |
| Risk-Based | IL-28B guided triple therapy | 15.1 | 3.5 | 19,277,327 | 266 | Dominated |
| Risk-Based | Universal triple therapy | 16.8 | 3.9 | 22,191,117 | 305 | Dominated |
| Birth-cohort | Standard therapy | 22.0 | 5.6 | 25,079,282 | 328 | Dominated |
| Birth-cohort | IL-28B guided triple therapy | 33.2 | 8.1 | 35,749,215 | 580 | $63,241 |
| Birth-cohort | Universal triple therapy | 35.8 | 8.7 | 40,316,174 | 647 | $68,084 |
| **Black Men** | |  |  |  |  |  |
| No Screening | Standard therapy | Reference | Reference | Reference | Reference | – |
| No Screening | IL-28B guided triple therapy | 9.6 | 2.4 | 9,384,635 | 169 | $55,560 |
| No Screening | Universal triple therapy | 10.2 | 2.6 | 10,652,151 | 181 | $104,557 |
| Risk-Based | Standard therapy | 12.8 | 3.8 | 26,159,688 | 77 | Dominated |
| Risk-Based | IL-28B guided triple therapy | 29.6 | 8.4 | 43,669,883 | 430 | Dominated |
| Risk-Based | Universal triple therapy | 30.7 | 8.7 | 46,078,726 | 455 | Dominated |
| Birth-cohort | Standard therapy | 32.2 | 9.6 | 55,846,516 | 220 | Dominated |
| Birth-cohort | IL-28B guided triple therapy | 59.8 | 17.3 | 84,420,949 | 848 | Dominated |
| Birth-cohort | Universal triple therapy | 61.6 | 17.9 | 88,372,059 | 892 | $109,264 |
| **Black Women** | |  |  |  |  |  |
| No Screening | Standard therapy | Reference | Reference | Reference | Reference | – |
| No Screening | IL-28B guided triple therapy | 4.8 | 1.0 | 5,824,720 | 97 | $59,948 |
| No Screening | Universal triple therapy | 5.2 | 1.1 | 6,612,111 | 104 | $114,255 |
| Risk-Based | Standard therapy | 5.3 | 1.5 | 15,486,098 | 24 | Dominated |
| Risk-Based | IL-28B guided triple therapy | 13.1 | 3.4 | 25,522,202 | 214 | Dominated |
| Risk-Based | Universal triple therapy | 13.7 | 3.6 | 26,899,832 | 227 | Dominated |
| Birth-cohort | Standard therapy | 14.3 | 4.0 | 31,550,745 | 64 | Dominated |
| Birth-cohort | IL-28B guided triple therapy | 27.1 | 7.3 | 47,557,195 | 397 | Dominated |
| Birth-cohort | Universal triple therapy | 27.9 | 7.5 | 49,776,718 | 420 | $136,783 |

ICER = incremental cost-effectiveness ratio; IL-28B = interleukin-28B; QALY = quality-adjusted life-year.

* F0 13%, F1 51%, F2 13 %, F3 10%, and F4 13%.

“Dominated” indicates that the strategy costs more and provides fewer benefits than another strategy or a combination of two strategies.

**Figure S2 Cost-effectiveness analysis A. Men B. Women**

**
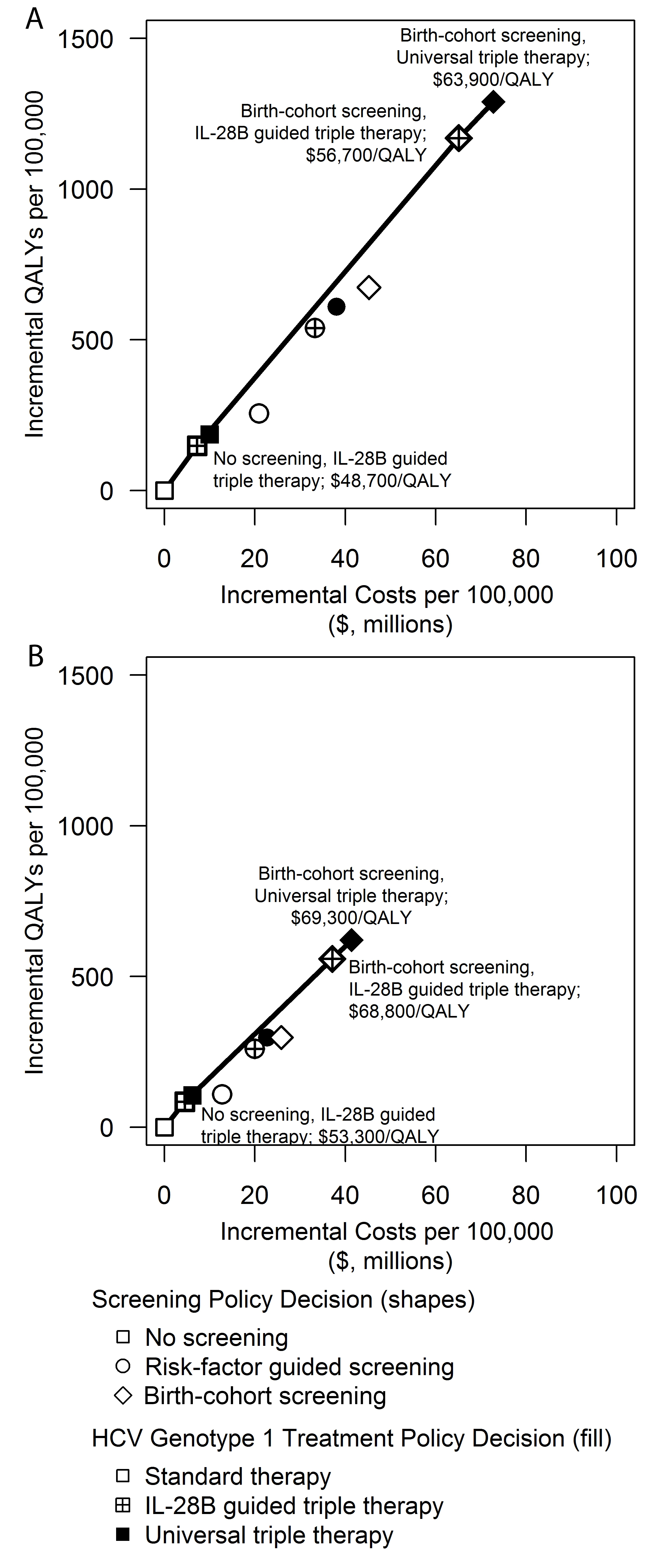
**

**Additional cost-effectiveness on limited strategy sets**

Not all technologies may be available in all places. We performed an analysis of the cost effectiveness of risk-based and birth-cohort screening if only standard therapy (and not triple therapy) were available. In this analysis, birth-cohort screening continued to provide better value than risk-based screening and compared to no screening, birth-cohort screening costs $73,365 per QALY gained. We also performed an analysis in which we considered that standard therapy and triple therapy are available treatment options, but that IL-28B genotyping is not available. In this analysis, we found that universal use of triple therapy costs $55,771 per QALY gained compared to standard therapy when there is no screening. Compared to this, birth-cohort screening with triple therapy costs $60,576 per QALY gained (Table S5).

**Table S5. Base case lifetime costs, health benefits (per 100,000), and incremental costs effectiveness ratio of selected screening and treatment strategies for a cohort of individuals who are currently 50 years of age.**

| **STRATEGY** | | **Per 100,000*** | | | |
| --- | --- | --- | --- | --- | --- |
| **Screening Strategy** | **Treatment Strategy** | **Incremental Cost ($)** | **Incremental QALY** | **ICER ($/QALY)** |  |
| **Standard therapy only** |  |  |  |  |  |
| No Screening | Standard therapy | Reference | Reference |  |  |
| Risk-Based | Standard therapy | 16,795,805 | 181 | Dominated |  |
| Birth-cohort | Standard therapy | 35,369,580 | 483 | $73,265 |  |
| **Standard and triple therapy** |  |  |  |  |  |
| No Screening | Standard therapy | Reference | Reference |  |  |
| No Screening | Universal triple therapy | 8,076,805 | 145 | $55,771 |  |
| Risk-Based | Standard therapy | 16,795,805 | 181 | Dominated |  |
| Risk-Based | Universal triple therapy | 30,282,373 | 450 | Dominated |  |
| Birth-cohort | Standard therapy | 35,369,580 | 483 | Dominated |  |
| Birth-cohort | Universal triple therapy | 56,843,606 | 950 | $60,576 |  |

* F0 13%, F1 51%, F2 13 %, F3 10%, and F4 13%. Population weighted average (white male 44%, white female 45%, black male 5%, black female 6%).

“Dominated” indicates that the strategy costs more and provides fewer benefits than another strategy or a combination of two strategies.

**Additional cost-effectiveness on triple therapy with telaprevir**

The reported triple therapy effectiveness used in the base-case analysis is similar to boceprevir. In the scenario analysis of telaprevir, we increased the overall probability of SVR to represent the effectiveness reported in the telaprevir ADVANCE (A New Direction in HCV Care: A Study of Treatment-Naive Hepatitis C Patients with Telaprevir) trial, white patients (SVR, 75%) and black patients (SVR, 61%). The result showed that the choice of the protease inhibitor for triple therapy does not change our main findings.

**Table S6. Triple therapy with telaprevir, lifetime costs, health benefits (per 100,000), and incremental costs effectiveness ratio of combined screening and treatment strategies for a cohort of individuals who are currently 50 years of age.**

| **COMBINED STRATEGIES** | | **Per 100,000*** | | | |
| --- | --- | --- | --- | --- | --- |
| **Screening** | **Treatment** | **Incremental Cost ($)** | **Incremental QALY** | **ICER ($/QALY)** |  |
| No Screening | Standard therapy | Reference | Reference | -- |  |
| No Screening | IL-28B guided triple therapy | 9,954,514 | 148 | Dominated |  |
| No Screening | Universal triple therapy | 14,159,353 | 200 | Dominated |  |
| Risk-Based | Standard therapy | 16,795,805 | 181 | Dominated |  |
| Risk-Based | IL-28B guided triple therapy | 33,549,149 | 459 | Dominated |  |
| Birth-cohort | Standard therapy | 35,369,580 | 483 | Dominated |  |
| Risk-Based | Universal triple therapy | 40,576,388 | 554 | Dominated |  |
| Birth-cohort | IL-28B guided triple therapy | 62,090,066 | 965 | $64,337 |  |
| Birth-cohort | Universal triple therapy | 73,285,951 | 1129 | $68,371 |  |

* Population weighted average (white male 44%, white female 45%, black male 5%, black female 6%) for fibrosis distribution: F0 13%, F1 51%, F2 13 %, F3 10%, and F4 13%. All incremental cost and QALY are compared to the reference.

ICER = incremental cost-effectiveness ratio; IL-28B = interleukin-28B; QALY = quality-adjusted life-year.

“Dominated” indicates that the strategy costs more and provides fewer benefits than another strategy or a combination of two strategies.

**Probabilistic Sensitivity Analyses**

**Figure S3. Cost-effectiveness acceptability curve**


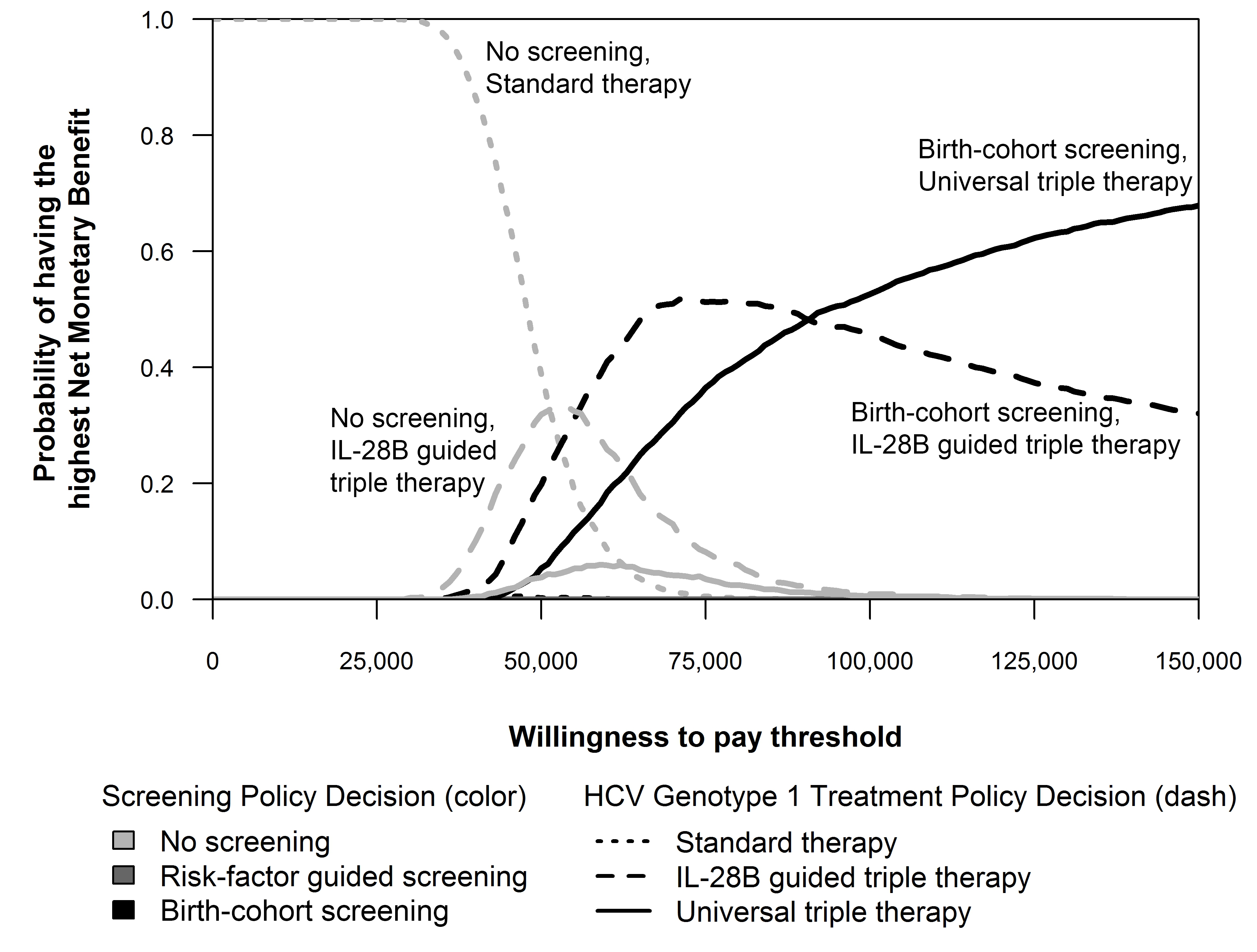


**Reference**

1. Liu S, Cipriano LE, Holodniy M, Owens DK, Goldhaber-Fiebert JD (2012) New protease inhibitors for the treatment of chronic hepatitis C: a cost-effectiveness analysis. Ann Intern Med 156: 279-290.

2. McHutchison JG, Lawitz EJ, Shiffman ML, Muir AJ, Galler GW, et al. (2009) Peginterferon Alfa-2b or Alfa-2a with Ribavirin for Treatment of Hepatitis C Infection. New England Journal of Medicine 361: 1027-1027.

3. Thompson AJ, Muir AJ, Sulkowski MS, Ge D, Fellay J, et al. (2010) Interleukin-28B Polymorphism Improves Viral Kinetics and Is the Strongest Pretreatment Predictor of Sustained Virologic Response in Genotype 1 Hepatitis C Virus. Gastroenterology 139: 120-U178.

4. Davis GL, Wong JB, McHutchison JG, Manns MP, Harvey J, et al. (2003) Early virologic response to treatment with peginterferon alfa-2b plus ribavirin in patients with chronic hepatitis C. Hepatology 38: 645-652.

5. Background Materials for Boceprevir Advisory Committee Division of Antiviral Products (DAVP). Available: <http://www.fda.gov/downloads/AdvisoryCommittees/CommitteesMeetingMaterials/drugs/AntiviralDrugsAdvisoryCommittee/ucm252341.pdf>. Accessed 15 May 2011.

6. Poordad F, McCone J, Bacon BR, Bruno S, Manns MP, et al. (2011) Boceprevir for Untreated Chronic HCV Genotype 1 Infection. N Engl J Med 364: 1195-1206.

7. Merck (2011) FDA Antiviral Drugs Advisory Committee Meeting Boceprevir Capsules (NDA 202-258) Briefing Document. Available: <http://www.fda.gov/downloads/AdvisoryCommittees/CommitteesMeetingMaterials/Drugs/AntiviralDrugsAdvisoryCommittee/UCM252343.pdf>. Accessed 15 May.

8. Vertex Pharmaceuticals (2011) Telaprevir 375-mg Film-Coated Tablet for the Treatment of Genotype 1 Chronic Hepatitis C, Antiviral Drugs Advisory Committee Briefing Document (Telaprevir NDA 201-917). Available: <http://www.fda.gov/downloads/AdvisoryCommittees/CommitteesMeetingMaterials/Drugs/AntiviralDrugsAdvisoryCommittee/UCM252562.pdf>. Accessed 15 May.

9. Birnkrant D, Team TR (2011) Advisory Committee Briefing Document for NDA 201-917 Telaprevir 375 mg tablets. Silver Spring: Department of Health & Human Services, Public Health Service, Food and Drug Administration. Available: http://www.fda.gov/downloads/AdvisoryCommittees/CommitteesMeetingMaterials/Drugs/AntiviralDrugsAdvisoryCommittee/UCM252561.pdf. Accessed 1 May 2011.

10. NATAP website. Levin J. Telaprevir in Combination with Peginterferon alfa-2a and Ribavirin in Genotype 1 HCV Treatment-Naïve patients: Final results of Phase 3 ADVANCE Study 2010; Boston, MA, Hynes Convention Center. Available: http://www.natap.org/2010/AASLD/AASLD_23.htm. Accessed 1 March 2011.
